# Differentiation of silent corticotroph pituitary neuroendocrine tumors (PitNETs) from non-functioning PitNETs using kinetic analysis of dynamic MRI

**DOI:** 10.1007/s11604-023-01420-3

**Published:** 2023-04-07

**Authors:** Taishi Amano, Tomohiko Masumoto, Daisuke Watanabe, Sodai Hoshiai, Kensaku Mori, Noriaki Sakamoto, Hiroyoshi Kino, Hiroyoshi Akutsu, Takahito Nakajima

**Affiliations:** 1 Department of Diagnostic and Interventional Radiology, University of Tsukuba Hospital, 2-1-1 Amakubo, Tsukuba, Ibaraki 305-8576 Japan; 2 Department of Diagnostic Radiology, Toranomon Hospital, Minato-ku, Tokyo, Japan; 3 Department of Diagnostic Pathology, University of Tsukuba Hospital, Tsukuba, Ibaraki Japan; 4 Department of Neurosurgery, University of Tsukuba Hospital, Tsukuba, Ibaraki Japan; 5 Department of Neurosurgery, Dokkyo Medical University, Shimotsuga-gun, Tochigi Japan

**Keywords:** Magnetic resonance imaging, Dynamic contrast enhancement, Pituitary neuroendocrine tumor, Silent corticotroph pituitary neuroendocrine tumor

## Abstract

**Purpose:**

Silent corticotroph pituitary adenomas (SCAs)/pituitary neuroendocrine tumors (PitNETs) are common non-functioning pituitary adenomas (NFAs)/PitNETs with a clinically aggressive course. This study aimed to investigate the ability of time-intensity analysis of dynamic magnetic resonance imaging (MRI) for distinguishing adrenocorticotropic hormone (ACTH)-positive SCAs and ACTH-negative SCAs from other NFAs.

**Materials and methods:**

We retrospectively evaluated the dynamic MRI findings of patients with NFAs. The initial slope of the kinetic curve (slope_ini_) obtained by dynamic MRI for each tumor was analyzed using a modified empirical mathematical model. The maximum slope of the kinetic curve (slope_max_) was obtained by geometric calculation.

**Results:**

A total of 106 patients with NFAs (11 ACTH-positive SCAs, 5 ACTH-negative SCAs, and 90 other NFAs) were evaluated. The kinetic curves of ACTH-positive SCAs had significantly lesser slope_ini_ and slope_max_ compared with ACTH-negative SCAs (*P* = 0.040 and* P* = 0.001, respectively) and other NFAs (*P* = 0.018 and *P* = 0.035, respectively). Conversely, the slope_ini_ and slope_max_ were significantly greater in ACTH-negative SCAs than in NFAs other than ACTH-negative SCAs (*P* = 0.033 and *P* = 0.044, respectively). In receiver operating characteristic analysis of ACTH-positive SCAs and other NFAs, the area under the curve (AUC) values for slope_ini_ and slope_max_ were 0.762 and 0748, respectively. In predicting ACTH-negative SCAs, the AUC values for slope_ini_ and slope_max_ were 0.784 and 0.846, respectively.

**Conclusions:**

Dynamic MRI can distinguish ACTH-positive SCAs and ACTH-negative SCAs from other NFAs.

## Introduction

Pituitary adenomas, described as pituitary neuroendocrine tumors (PitNETs) in the 5th edition of the World Health Organization (WHO) Classifications (2021 WHO Classification of Central Nervous System Tumors and 2022 WHO Classification of Endocrine and Neuroendocrine Tumors) [1], are the most common intracranial endocrine tumors and are broadly classified clinically into functioning adenomas that produce hormones and non-functioning adenomas (NFAs) that do not produce hormones. NFAs account for approximately 15–54% of pituitary adenomas [2, 3]. Unlike functional pituitary adenomas, NFAs lack hormonal secretory symptoms and are usually asymptomatic; however, as the tumor grows, they present with symptoms such as abnormal vision, hypopituitarism, and headache. NFAs are often detected incidentally in imaging studies performed for other purposes. According to the 4th edition of the WHO Classification of Endocrine Tumors, NFAs can be categorized into silent gonadotroph adenomas (SGAs), silent corticotroph adenomas (SCAs), silent somatomammothyrotroph adenomas, null cell adenomas, plurihormonal NFAs, and double NFAs [4]. In the 5th edition of the WHO Classification of Endocrine and Neuroendocrine Tumors, nonfunctioning PitNETs are classified according to their frequency as silent gonadotroph tumors, silent corticotroph tumors, silent immature PIT1-lineage tumors, null cell tumors, and other tumors [5]. This classification is based on the immunohistochemical expression of pituitary hormones and transcription factors, making preoperative subtype classification difficult using clinical or hematological examinations.

Specific subtypes of pituitary adenomas often have a clinically aggressive course and are referred to as aggressive adenomas. These include sparsely granulated somatotroph adenomas, lactotroph adenomas in men, SCAs, Crooke’s cell adenomas, and plurihormonal pituitary-specific positive transcription factor 1 (Pit-1) adenomas. SCAs are the most common aggressive adenoma among NFAs [6], expressing the t-box pituitary transcription factor (T-pit). SCAs are subclassified into adrenocorticotropic hormone (ACTH)-positive and ACTH-negative SCAs. SCAs are highly proliferative and invasive, with a high frequency of late multiple recurrences in young patients [6, 7]. They are resistant to postoperative irradiation [8] and can transform into functioning corticotroph adenomas [9]. Given the progressive course of these SCAs, preoperative imaging distinction between SCAs and other NFAs would help determine a course of follow-up and treatment. Cazabat et al. used non-contrast magnetic resonance imaging (MRI) to distinguish SCAs from other NFAs and reported that SCAs have significantly more multiple microcysts than other NFAs [10]. Discrimination of NFAs using radiomics has also been reported [11, 12]. Dynamic MRI has been used for histological differentiation of pituitary adenomas, including for distinguishing ACTH-producing pituitary adenomas from non-functioning pituitary adenomas [13] and distinguishing growth hormone-producing pituitary adenomas and prolactin-producing pituitary adenomas from other pituitary adenomas [14]. However, the effectiveness of dynamic MRI in discriminating SCAs from NFAs has not been evaluated.

Past studies have shown that the steepest slope of the time-intensity curve obtained from dynamic MRI of tumors correlates with the vascular density and degree of malignancy of the tumor tissue [15–17]. Several empirical mathematical models have been developed to describe the behavior of contrast uptake by tumors. Jansen et al. developed a modified empirical mathematical model (EMM) for using dynamic MRI to differentiate between benign and malignant lesions in the breast [18]. Fitting the dynamic MRI kinetic curve to an EMM allowed parameters such as the initial slope to be calculated. EMM analysis by Mori et al. also revealed that the initial slope of the kinetic curve was highly correlated with the microvessel density (MVD) of invasive breast cancer [19]. In our institution, dynamic MRI is performed as a preoperative evaluation in all cases of pituitary adenomas, regardless of size. We have empirically found that dynamic contrast enhancement patterns in pituitary adenomas vary from a rapid to a gradual enhancement pattern. If these enhancement patterns are associated with NFA subtypes, dynamic MRI findings may be useful in predicting these subtypes. Histologic vascular features of pituitary adenomas vary by subtype, and the vascular density of ACTH-positive SCAs is known to be less than that of other NFAs [20]. This study aimed to examine the usefulness of time-intensity curve analysis of dynamic MRI for histologically differentiating ACTH-positive SCAs and ACTH-negative SCAs from other NFAs.

## Materials and methods

### Patients

This retrospective study was approved by the appropriate institutional review board and was conducted according to the tenets of the Declaration of Helsinki. The requirement for informed consent waived. Patients who underwent high temporal resolution dynamic MRI (11–13.2 s) at our hospital and were diagnosed histopathologically with pituitary adenomas by surgery were included. NFAs were defined as pituitary adenomas lacking pituitary hormone hypersecretion. Immunohistochemical staining was performed to detect pituitary hormones using monoclonal antibodies against ACTH, prolactin, growth hormone, luteinizing hormone, follicle-stimulating hormone, and thyroid-stimulating hormone. To identify ACTH-negative SCAs, antibodies against T-pit (TPIT, HPA072686; Atlas Antibodies) were used to test tumors that were negative for all hormones or were co-positive for ACTH and other hormones. Given that NF macroadenomas can cause mild hyperprolactinemia due to pituitary stalk compression, prolactin-positive macroadenomas with blood prolactin levels above normal but less than 200 ng/ml were considered NFAs. MR images of NFAs were obtained from 115 patients between August 2011 and March 2022.

### MRI parameters

MR images were acquired on an Achieva 1.5 T, Achieva 3.0 T system, or Ingenia 3.0 T system (all from Philips Healthcare; Best, the Netherlands). Imaging sequences included a sagittal and coronal T2-weighted fast spin–echo sequence (section thickness, 3.0 mm; slice gap, 0.3 mm; field of view [FOV], 160 mm; TE, 80–90 ms; TR, 3000 ms; flip angle, 90°). Dynamic MRI images were acquired using a sagittal and coronal T1-weighted fast spin–echo sequence (section thickness, 3.0 mm; slice gap, 0.3 mm; FOV, 160 mm; TE, 12 ms; TR, 500–600 ms; flip angle, 80–90°). A bolus injection of 0.1 mmol/kg of the contrast agent (gadoteridol; ProHance; Bracco Eisai, Tokyo) was administered at 2.5 ml/s. A dynamic MRI scan was performed after contrast injection with 15 scans at 11-, 12-, or 13.2-s intervals.

### Analysis of lesion enhancement using dynamic MRI

Dynamic MR signal intensity values were measured in a circular or oval region of interest (ROI) where the highest signal increase was observed within each lesion. The ROI area was set to be at least 25 mm^2^. The MR signal intensity as a function of time in the ROI of each phase in dynamic MRI was defined as S(t). S_pre_ was the signal value in the ROI before the arrival of the contrast agent. The time when the signal value in the ROI reached 1.2 times greater than S_pre_ was defined as the point when the contrast agent arrived at the tumor. The signal change after contrast injection was then calculated as follows:

$$\Delta S \, = \, (S(n) - S_{{{\text{pre}}}} )/S_{{{\text{pre}}}}$$

where S(n) is the signal value in the ROI at the nth phase after the arrival of the contrast agent. The following modified EMM developed by Jansen et al. [18] was used as an approximation function to represent contrast uptake and washout of the tumor:

$$\Delta S(t) \, = \, A \, \times \, (1 - e^{ - at} ) \, \times \, e^{ - bt}$$

where A is the upper limit of the signal intensity, α (min^−1^) is the rate of signal increase, and β (min^−1^) is the rate of the signal decrease during washout. The accuracy of fit for the *R*^2^ parameter was calculated for each lesion.

As a diagnostic parameter, the initial slope of the approximate kinetic curve (slope_ini_) was calculated as the product of the uptake rate *α* and the amplitude of enhancement *A*:

$${\text{slope}}_{{{\text{ini}}}} = A\alpha$$

From the same ROI, the maximum slope (slope_max_) of the measured kinetic curve was calculated as another diagnostic parameter [15–17]. Slope_max_ is the maximum value of slope (*n*) shown below:

$${\text{slope}}\;(n) \, = \, (S(n + 1) \, - \, S(n)) \, / \, (t(n + 1) \, - \, t(n))$$

where *S*(*n*) is the signal value of the ROI in the nth phase after the arrival of the contrast agent, and *t*(*n*) is the time of the *n*th phase. We developed programs using Python 3.9.16 to draw kinetic curves and retrieve diagnostic parameters and ran these on a 2.9 GHz machine with 16 GB of RAM (MacBook Pro 2017).

In a previous report, the presence of multiple microcysts inside the tumor on MRI was useful in discriminating SCAs from NFAs [10]. Therefore, we evaluated the presence of multiple microcysts within tumors to compare the usefulness of this finding with our results. Following the previous study [10], multiple microcysts were defined as those that were < 3 mm in diameter and covered > 25% of the solid portion of the tumor. The pituitary tumor size was measured as the maximum diameter (mm) on sagittal or coronal T2-weighted images.

### Statistical analysis

The slope_ini_ and slope_max_ were analyzed to differentiate between SCAs and other NFAs. As pathological subtypes, ACTH-positive SCAs and ACTH-negative SCAs were analyzed to distinguish them from other NFAs. The tumor size was also analyzed to determine its relationship with pathological subtypes. As these variables were non-normally distributed, statistical analyses were performed using the Mann–Whitney *U* test. Statistical significance was set at *P* < 0.05. Bonferroni correction was applied for multiple comparisons. The receiver operating characteristic (ROC) curve was used to assess diagnostic performance. The presence of multiple microcysts in the tumor was evaluated to differentiate SCAs from other NFAs, and the sensitivity and specificity of the presence of multiple microcysts to predict SCAs were determined. We determined the cutoff values of slope_ini_ and slope_max_ in dynamic MRI that yielded the greatest sensitivity and specificity for predicting the presence of SCA. The usefulness of these values was compared with that of the microcyst assessment method [10]. All statistical analyses were performed using SPSS version 28.0.

## Results

**Fig. 1:**
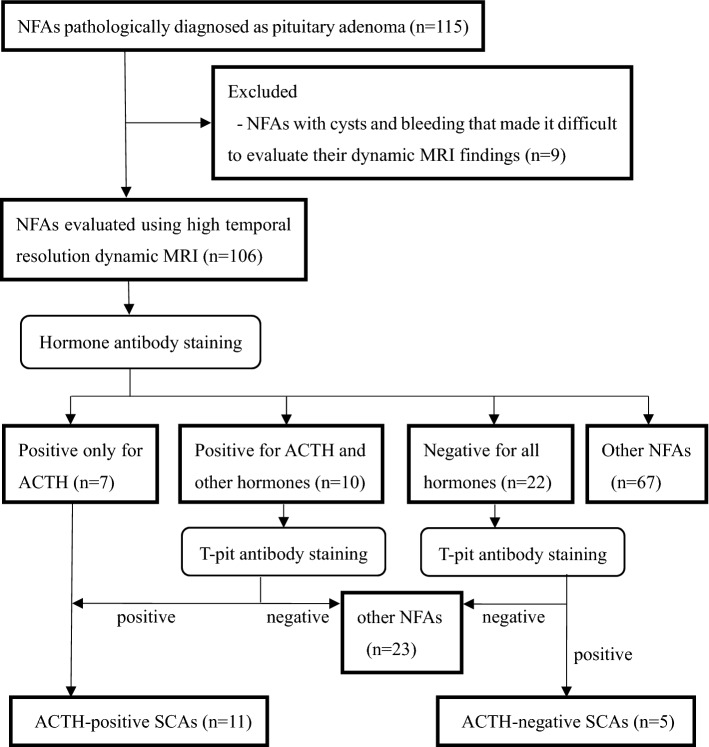
Flowchart of the patient enrollment process. *NFA* non-functioning adenoma, *ACTH* adrenocorticotropic hormone, *SCA* silent corticotroph adenoma

Figure 1 summarizes the patient enrollment process. Among the 115 patients with NFAs diagnosed pathologically as pituitary adenoma, we excluded 9 patients with severe cystic degeneration and hemorrhage, which impeded the evaluation of contrast images. Among the remaining 106 patients, 11 patients with ACTH-positive SCA, 5 patients with ACTH-negative SCA, and 90 patients with other NFAs were diagnosed pathologically using hormone antibody staining and T-pit antibody staining.

Table 1 summarizes the dynamic MRI findings. The modified EMM accurately fit the dynamic MRI time-intensity curves, with a mean value and standard deviation value of 0.97 ± 0.04 for *R*^2^, the goodness-of-fit parameter. Kinetic curves were drawn and diagnostic parameters were obtained for each case in less than 1 s of processing time. The respective mean values for slope_ini_ and slope_max_ were 0.067 and 0.044 for ACTH-positive SCAs, 0.218 and 0.078 for ACTH-negative SCAs, and 0.109 and 0.057 for the other NFAs, respectively. Tumor size was not significantly different among these pathologic subtypes.

**Table 1:** MRI findings of non-functioning adenomas

NFA type	Number of cases	Sex (M:F)	Age (years)	Maximum diameter (mm)	Number of cases with microcysts	Slope_ini_	Slope_max_
ACTH-positive SCAs	11	3:8	59 (± 16)	28.5 (± 11.0)	3	0.067 (± 0.024)	0.044 (± 0.009)
Other NFAs	90	56:34	59 (± 13)	29.8 (± 10.0)	8	0.109 (± 0.055)	0.057 (± 0.018)
ACTH-negative SCAs	5	0:5	50.0 (± 9)	33.8 (± 15.3)	3	0.218 (± 0.123)	0.078 (± 0.016)

Data are presented as the means (± standard deviations)

*NFA* non-functioning adenoma, *ACTH* adrenocorticotropic hormone, *SCA* silent corticotroph pituitary adenoma

**Fig. 2:**
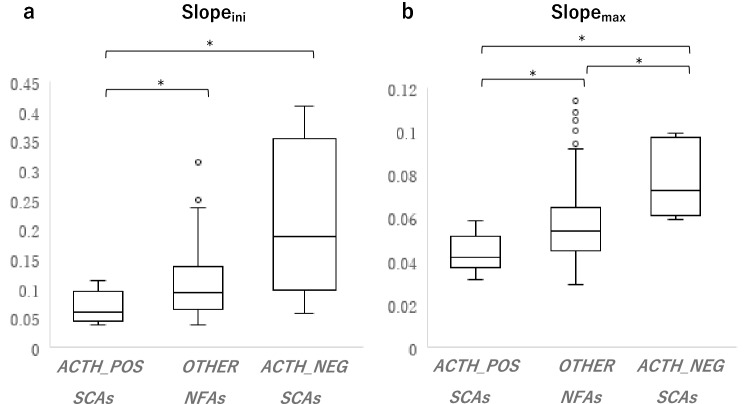
Comparison of slope_ini_ and slope_max_ for each NFA (*ACTH_POS SCAs* ACTH-positive SCAs, *ACTH_NEG SCAs* ACTH-negative SCAs). The Bonferroni correction is applied for multiple comparisons. **a** Box-and-whisker plots showing that the slope_ini_ is significantly less in ACTH-positive SCAs than in ACTH-negative SCAs and other NFAs (*P* = 0.040 and *P* = 0.018, respectively). The slope_ini_ is greater in ACTH-negative SCAs than in other NFAs, but the difference is not statistically significant (*P* = 0.129). ACTH-negative SCAs have a statistically significant greater slope_ini_ compared to NFAs other than ACTH-negative SCAs (*P* = 0.033). **b** The slope_max_ is significantly less in ACTH-positive SCAs than in ACTH-negative SCAs and other NFAs (*P* = 0.001 and *P* = 0.035, respectively). In contrast, the slope_max_ is significantly greater in ACTH-negative SCAs than in other NFAs (*P* = 0.044). *Statistically significant (*P* < 0.05)

Figure 2 shows box-and-whisker plots comparing the slope_ini_ and slope_max_ for each subtype of NFA. The slope_ini_ was significantly less in ACTH-positive SCAs than in ACTH-negative SCAs and other NFAs (*P* = 0.040 and *P* = 0.018, respectively). In contrast, the slope_ini_ tended to be greater in ACTH-negative SCAs than in other NFAs. The difference between ACTH-negative SCAs and other NFAs was not statistically significant (*P* = 0.129) (Fig. 2a); however, ACTH-negative SCAs had a significantly greater slope_ini_ value compared to NFAs other than ACTH-negative SCAs (*P* = 0.033). The slope_max_ was also significantly less in ACTH-positive SCAs than in ACTH-negative SCAs and other NFAs (*P* = 0.001 and *P* = 0.035, respectively). In contrast, the slope_max_ was significantly greater in ACTH-negative SCAs than in other NFAs (*P* = 0.044) (Fig. 2b).

**Fig. 3:**
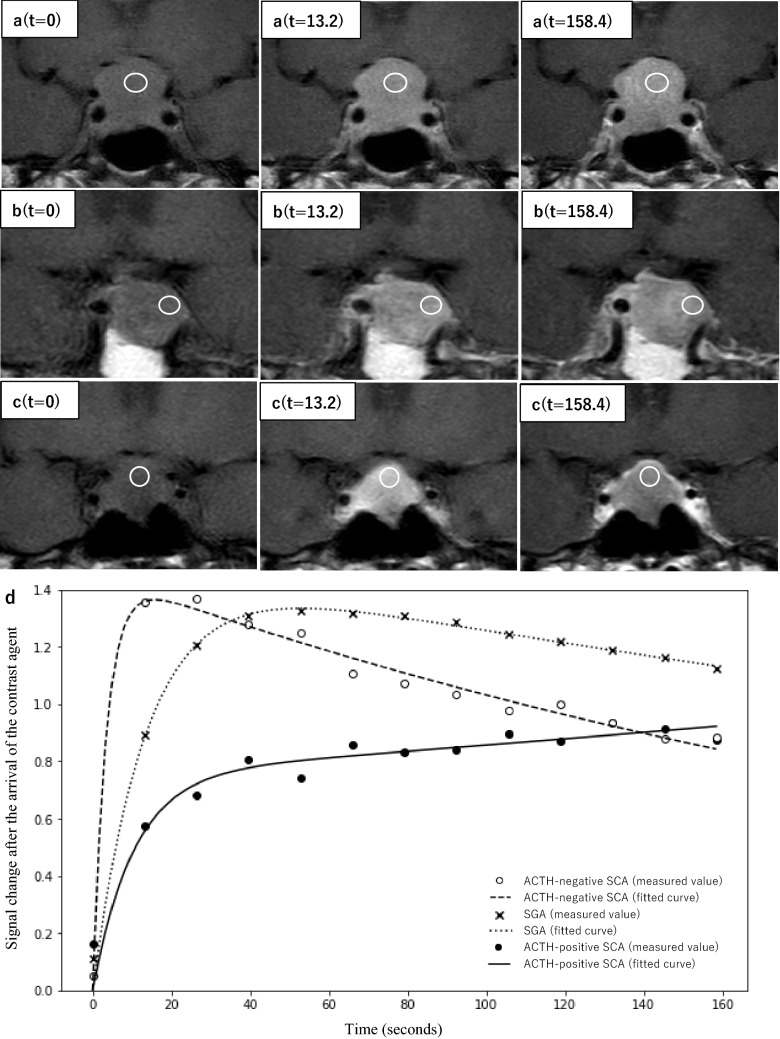
The initial dynamic MRI phase and late phase images. **a** A 36-year-old man with adrenocorticotropic hormone (ACTH)-positive silent corticotroph pituitary adenoma (SCA). **b** A 77-year-old woman with silent gonadotroph adenoma (SGA). **c** A 46-year-old woman with ACTH-negative SCA. Dynamic MR signal intensity values are measured in a circular or oval region of interest (ROI) where the highest signal increase is observed within each lesion. The ROI is copied to all the phases (white circle). The ACTH-positive SCA case shows a relatively more-gradual contrast in the early phase than the SGA case (**a**, **b**). In contrast, the ACTH-negative SCA case shows relatively more-rapid contrast in the early phase than the SGA case (**b**, **c**). The time-intensity curve of this case is shown (**d**). For the ACTH-positive SCA case, the measured values are plotted as black circles, and the fitted curve is shown as a solid line; the time-intensity curve has a more gradual rise than the SGA case (measured values are plotted as a cross; the fitted curve is shown as a dotted line). For the ACTH-negative SCA case, the measured values are plotted as white circles, and the fitted curve is shown as a dashed line; the time-intensity curve has a more rapid rise than the SGA case

Examples of the dynamic MR images and a time-intensity curve of ACTH-positive SCA (with lesser slope_ini_ and slope_max_), ACTH-negative SCA (with greater slope_ini_ and slope_max_), and SGA are shown in Fig. 3. A case of ACTH-positive SCA with lesser slope_ini_ and slope_max_ (Fig. 3a, d) showed a relatively gradual contrast effect in the early stages of dynamic MRI. In contrast, a case of ACTH-negative SCA with greater slope_ini_ and slope_max_ (Fig. 3c, d) showed a relatively rapid contrast effect in the early stages of dynamic MRI.

**Fig. 4:**
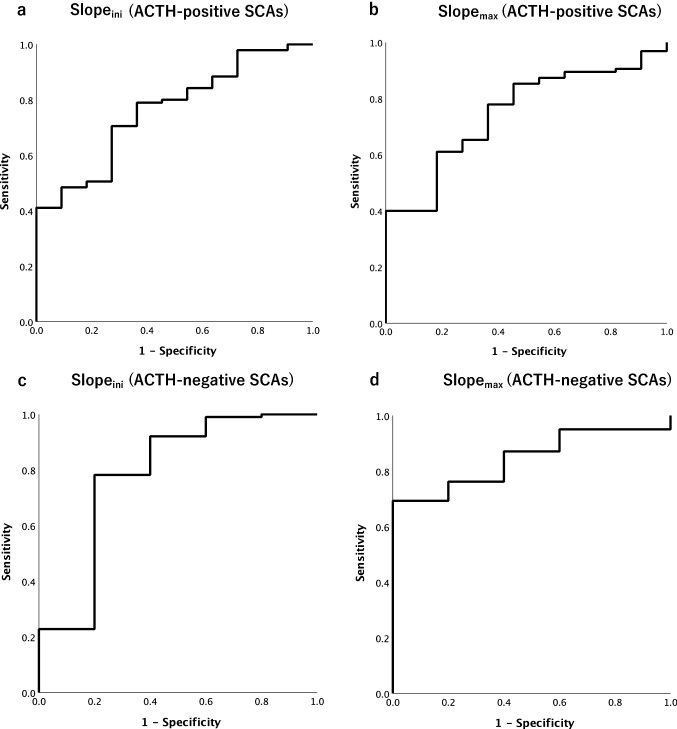
ROC curves of the slope_ini_ and slope_max_ for predicting ACTH-positive SCAs and ACTH-negative SCAs. For predicting ACTH-positive SCAs, the areas under the ROC curves for slope_ini_ (**a**) and slope_max_ (**b**) are 0.762 and 0.748, respectively. For predicting ACTH-negative SCAs, the areas under the ROC curves for slope_ini_ (**c**) and slope_max_ (**d**) are 0.784 and 0.846, respectively

ROC curves focused on the differential diagnosis between ACTH-positive SCAs and other NFAs, and between ACTH-negative SCAs and other NFAs were analyzed using data from slope_ini_ and slope_max_ (Fig. 4). For predicting ACTH-positive SCAs, the area under the curve (AUC) values for slope_ini_ and slope_max_ were 0.762 and 0.748, respectively (Fig. 4a, b). For predicting ACTH-negative SCAs, the AUC values for slope_ini_ and slope_max_ were 0.784 and 0.846, respectively (Fig. 4c, d). For predicting ACTH-positive SCAs, the cutoff values for maximum sensitivity and specificity in the ROC analysis were 0.074 and 0.051 for slope_ini_ and slope_max_, respectively; the predictive ability for ACTH-positive SCAs at each cutoff value had 71% sensitivity and 73% specificity for slope_ini_ and 61% sensitivity and 82% specificity for slope_max_. For predicting ACTH-negative SCAs, the cutoff values for maximum sensitivity and specificity in the ROC analysis were 0.138 and 0.059 for slope_ini_ and slope_max_, respectively; the predictive ability for ACTH-negative SCAs at each cutoff value had 78% sensitivity and 80% specificity for slope_ini_ and had 69% sensitivity and 100% specificity for slope_max_. Multiple microcysts were present in 27% (3/11) of ACTH-positive SCAs, 60% (3/5) of ACTH-negative SCAs, and 9% (8/90) of the other NFAs. The sensitivity and specificity for predicting ACTH-positive and ACTH-negative SCAs were 27% and 88% and were 60% and 89%, respectively.

## Discussion

Our findings indicate that dynamic MRI can identify ACTH-positive SCAs and ACTH-negative SCAs in NFAs. Compared to other NFAs, ACTH-positive SCAs were found to have a more gradual rise in the time-intensity curve, and ACTH-negative SCAs were found to have a more rapid rise in the time-intensity curve. ROC analysis for differentiating ACTH-positive SCAs from other NFAs showed AUC values of 0.762 and 0.748 for slope_ini_ and slope_max_, respectively. ROC analysis for differentiating ACTH-negative SCAs from other NFAs showed AUC values of 0.784 and 0.846 for slope_ini_ and slope_max_, respectively.

In previous studies on breast, rectal, and bladder tumors, the steepest slope of the dynamic MRI time-intensity curve correlated significantly with the MVD [15–17]. Mori et al. [19] reported that the initial slope of an EMM-based approximation curve of the time-intensity curve correlated significantly with the MVD in breast cancer. Turner et al. [20] evaluated MVD in pituitary adenomas by labeling with endothelial markers using three different antibodies; they reported that ACTH-positive SCA was the least vascular type of NFA and showed a significant difference in endothelial markers using antibodies against CD31. These reports are consistent with our data showing that ACTH-positive SCAs have lesser values for slope_ini_ and slope_max_ than other NFAs. Although the MVDs of malignant tumors generally correlate with the grade and tumor stage, tumor aggressiveness in pituitary adenomas is known not to correlate with markers for angiogenesis or vasculogenesis [21]. Vascularity is not higher even in pituitary carcinomas than in normal pituitary tissue [21]. These reports are consistent with the tendency of ACTH-positive SCAs to have low MVD, despite having higher proliferative and invasive potentials, higher recurrence rate, and stronger resistance to radiotherapy than other NFAs. Guo et al. [13] reported a lesser dynamic MRI pre-peak slope (referring to the measure of the tissue perfusion rate or the rate of reaching peak intensity in the enhancement curve) in ACTH-producing pituitary adenomas than in NFAs. Turner et al. [20] reported lower levels of histological markers of angiogenesis in ACTH-producing tumors than in NFAs. Considering that both ACTH-producing pituitary adenomas and ACTH-positive SCAs stain immunopositively for ACTH and share low levels of histological markers of angiogenesis, it seems reasonable that they would show similar results on dynamic MRI findings. In contrast, ACTH-negative SCAs had greater slope_ini_ and slope_max_ than ACTH-positive SCAs and other NFAs. However, to the best of our knowledge, the levels of MVD and angiogenic markers in ACTH-negative SCAs could not be confirmed. MVD or levels of histological markers of angiogenesis may be greater in ACTH-negative SCAs than in other NFAs.

The incidence of microcysts in this study was 27% for ACTH-positive SCA and 60% for ACTH-negative SCA, which are greater than those of other NFAs (9%) and in agreement with the results of Cazabat et al. [10]. However, the sensitivity and specificity of predicting SCA with and without microcysts in this study were 38% and 91%, respectively, somewhat lower than the reported 76% sensitivity and 95% specificity [10]. Consistent with the results of Zhang et al. [22], the usefulness of microcysts to discriminate between SCA and other NFAs was more remarkable in ACTH-negative SCA (60% sensitivity and 89% specificity) than in ACTH-positive SCA (27% sensitivity and 88% specificity). The predictive ability for ACTH-negative SCA at the time of maximum sensitivity and specificity on dynamic MRI in this study had 69% sensitivity and 100% specificity for slope_max_, which are greater than the 60% sensitivity and 89% specificity of the conventional assessment method that used the presence of microcysts.

This study was conducted in a single center with a standard setup and long experience in pituitary adenoma practice. However, this study has several limitations. One is the combined use of different imaging MRI systems. In addition, this is a retrospective study, and the number of ACTH-negative pituitary adenomas is insufficient: only five cases. Further large prospective multicenter studies are needed to validate our results.

## Conclusions

Dynamic MRI can be used to distinguish ACTH-positive and ACTH-negative SCAs. Compared with other NFAs, ACTH-positive SCAs have a more gradual rise in the time-intensity curve, whereas ACTH-negative SCAs have a more rapid rise in the time-intensity curve. Further extensive prospective evaluations should be performed to confirm our findings.
